# Selections and Abstracts

**Published:** 1903-01

**Authors:** 


### SELECTIONS AND ABSTRACTS.

### Paralysis and Damage Suits.*

The commercial spirit of the age has forced a new and arduous duty upon the man of medicine. The assessment of damages for settlement of claims for the immediate and remote consequences of personal injury has become one of the most common and acute of the duties of the medical as well as the legal jurist.

Our knowledge of the effects of trauma upon the nervous system has become more complete and rounded out by the observations of our medical predecessors, who, though often at fault because of hasty conclusions, left to us a heritage of facts, statistics, and observations which now enables us to complete the task which they found was not bounded by the horizon of one life. The optimist says all is to some good.

No book ever aided the malingerer as Erichsen's Spinal Concussion. It is equally true that no book was ever produced which caused so much controversy; and in controversy lies truth and discovery. The railway companies of the world fought its conclusions and expended large sums maintaining and encouraging the idea that *all* was fraud. But truth, even when half error, has remarkable vitality, and while the pathology of Erichsen is now obsolete and a majority of his conclusions rejected, yet considering the time of his work it was and still remains a remarkable and epoch-making book.

The undying zeal of controversy, stimulated by the best of medical and legal talent arrayed on either side, has done much to elucidate, and is still doing much to clear up, the misty subject. That truth lies between the extremes seems evident from the decided modifications of the views of some of the radical of both parties.

---

*By Charles J. Aldrich, M.D., Cleveland, Ohio, Lecturer on Clinical Neurology and Anatomy of the Nervous System, College of Physicians and Surgeons; Neurologist to the Cleveland General Hospital and Dispensary; Neurologist to the Cleveland City Hospital.

It is of interest to note that that new factor, born of commercialism and controversy—the Claim Adjuster--does not hesitate to recognize the morbid state (hysteria) as a result of traumatism, be the trauma slight or great, and to advise his employers to settle outside of court.

Medical men have been extremely reluctant to acknowledge that rare cases of paralysis (hysterical) were promptly cured by the self-styled "healers," whose nearest approach to purity was the 14-caret gold pieces extracted from the deluded but deservedly grateful patient. Then why should we show surprise at the unbelief of our legal brother who witnesses the supposed paralytic (truly hysterical) forget his crutches in the exuberance of joy at winning his case and collecting his damages?

With few exceptions the medical man has come to recognize these cases, and no longer insults the patient or stultifies himself by crying *fraud.* But many railway surgeons have closed their eyes to the facts, and labor long to convince the world that a *traumatic neurosis* is but artful fraud, and its victim a shrewd malingerer of the deepest dye.

Railway surgeons as a class have made the mistake of not investigating these cases from the dispassionate standpoint of the neurologist, who has long known that these affections, while pathological frauds, are true clinical entities, and has demonstrated by undeniable statistics that the prognosis is much more grave than the railway surgeon would have us believe. Indeed, railway surgeons generally have been very slow to appreciate these investigations, but have been very quick to denounce them without due consideration. As evidence of this fact I wish to cite the case of the celebrated Freeman family, the story of which is told in a pamphlet that has been widely circulated among claim adjusters and railway surgeons, entitled "Paralysis as a Fine Art." Now I submit that any one who would advise or countenance the payment of one cent of damages to settle the claims of such self-evident, thinly-tissued frauds as this family of blackmailers has certainly not been very careful of his study of the nervous system in relation to the very important question of the detection of the malingerer. It is surprising that in the late book of Pearce Bailey on Accident and Injury he should call this pamphlet "interesting and cleverly written," implying his indorsement of the title of the pamphlet. If all examples of fine art were to be measured by such crude tests as seem to have been used in the examination of the various members of the Freeman family, I am quite certain that our amateurs need have little fear of sending their daubs to the Royal Academy. The whole pamphlet from beginning to end was evidently written by a man who knew almost nothing of hysteria, and very little regarding the organic palsies, or he would not parade the ignorance of his colleagues by chronicling in printers' ink the details of the examination made to determine the question of organic injury, hysteria, or malingering.

The company's physician speaks of the case in the following words: "Tenderness to pressure and percussion over the lumbar and dorsal vertebræ. Can't stand, half reclining posture, all of the time evidencing great agony. When I stuck pins into her feet and legs, and touched them with my hands, she declared she could not feel any sensation, and I could not surprise her into any painful expression."

We have in this report no determination of the condition of any of the sphincters, no adequate tests to determine their continence or incontinence; no tests or charts furnished showing the areas of anesthesia, no comparison made or attempted to determine the character of the anesthesia; whether central, spinal, peripheral, hysterical, or simulated.

*Simulation can only become a systematized occupation or means of livelihood through the ignorance of the examining surgeon.* Thorough, careful, painstaking investigation will invariably reveal the most artful malingerer. Oppenheim, Dana, Putnam, Bailey and Knapp, and in fact all of the great men who have made a study of the traumatic neuroses, are convinced of this fact.

There seems to be on the part of various writers a very wide difference as to the frequency of simulation. The oldest writers, of whom Schultze is a type, regarded simulation as very common, but that was in the days when hysteria and neurasthenia were but little understood. On the other hand, Oppenheim, Putnam, Knapp, and others regard it as comparatively infrequent, Oppenheim placing simulation as low as four per cent. Of course this is not intended to apply to the exaggeration of symptoms so common to the hysteriac and neurasthenic, which we all know is constant not only in litigation but in non-litigation cases.

When confronted by a case similar to that of Fannie Freeman, what shall the surgeon do, and what must he know to determine the question "Is she paralyzed or is she malingering?" The surgeon examining her said: "She complained of paralysis of motion and sensation from the waist downward, and an inability to control the bladder and rectum." "Tenderness to pressure and percussion over the lumbar and dorsal vertebræ" had little or nothing to do with the determination of this question. The fact that she said she could neither stand, walk, nor sit except when supported, and that she claimed to suffer agony during this process, is merely her personal statement and has nothing to do with the real truth of the proposition. The simple fact that she had sufficient control of her sensations to stand having a pin thrust into her feet and legs, and her bare statement that she felt neither punctures nor the touch of his hand, throws absolutely no light upon the question, unless the areas over which the anesthesia and the analgesia existed are carefully mapped out, and placed in the class of anesthesiæ to which they belong—be it a stocking anesthesia or the functional type, a unilateral anesthesia confined to the peripheral distribution of a nerve, or the symmetrical bilaterial anesthesia following the segmental type due to a lesion of a certain level of the cord.

Be this as it may, his prognosis was given as unfavorable, and Fannie got $450 and the surgeon his fee, and is probably still retained by the company that was mulcted by his lack of thorough examination. Now Fannie Freeman may have possessed an unfaltering will. She may have possessed, like many degenerates of her class, a degree of analgesia which is more or less common among degenerate females, and sufficient to have never been surprised into an expression of pain by pin-thrusts, but she certainly did not possess the knowledge nor power to defeat the result of some certain tests in relation to the sphincters, reflexes, circulation, and sensation. Had the anus been exposed to strong light, and, without her knowledge as to what was to be done, a feather quickly stroked over the skin about the anus, she would certainly and invariably have manifested a distinct contraction of the levator and sphincter muscles, demonstrating the falsity of her assurances that her rectal sphincter was incompetent. If this test fails, the finger without anointing should be quickly thrust into the anus, when neither she nor any one else, however great their will-power, could prevent the contraction of the sphincters upon the intruding finger. If the paralysis is actual, the laxity of the sphincters will demonstate the fact beyond cavil. If the examining surgeon had carefully counted her pulse previous to palpating her tender spine (?), her pulse-rate would have been accelerated if her statements were facts. He should have seated her in a good light and watched closely the pupils, which would almost certainly have dilated if pressure on her vertebræ gave her much suffering.

A paraplegia or a paralysis of the lower limbs without loss of bladder or rectal control is almost invariably due to hysteria or simulation. When the simulator insists that his bladder and rectum are paralyzed, he renders his case particularly susceptible of detection. We find nothing in the description of Fannie Freeman's case as to whether it was demonstrated that her clothing or the bed upon which she lay was saturated with the urine of an incontinent or overflowing bladder. We find nothing to tell us whether a catheter or bulbous sound was used to determine if the vesical sphincters contracted upon them.

Unfortunately for the money-loving Fannie, upon her next attempt to mulct the railway company they sent the same physician that had examined her before, who, to use the words of the pamphlet, "this time recognized the fraud, notwithstanding her persistent assurances that this was her first accident."

The subject of the detection of the malingerer is so extremely wide that it is impossible to compass its discussion in this paper. The few remaining remarks will be made in an attempt to show how, by systematic examination, it is always possible to detect one who simulates a spinal paralysis. This seems to be the most frequent form of paralysis that the simulator attempts. *To sham a pathological sham is a great undertaking.* Besides, if it is sham, why not sham the real thing and not its pathological counterfeit—hysteria? It requires no mean order of mind to be a successful malingerer. It requires no small amount of medical knowledge to be able to imitate a paraplegia of spinal origin, but I am satisfied that to be a successful malingerer and imitate a hysterical neurosis requires a mind and a knowledge almost beyond conception.

Given a malingerer with great self-control, power of imitation, and thorough knowledge of the symptoms of a genuine lesion of the spinal cord, and yet he cannot deceive a careful examiner. He might be able to feign areas characteristic of a lesion of a certain spinal level; he might cause grave doubts to arise in the mind of the examiner as to the integrity of his vesical and rectal sphincters; but he could not control the ascent of the testicle when the scrotum or skin of the inside of the thigh was smartly pinched, and it would be almost impossible for him to prevent muscular contraction incident to an unexpected titillation of the soles of the feet. (Of course, it is understood that during this examination he is blindfolded.) Neither could he give any sort of imitation of increased knee-jerk, and least of all ankle clonus. The most assiduous practice will not admit of any one producing the rhythmical to-and-fro movement of an ankle clonus that must develop sooner or later in a patient who has suffered a transverse lesion of the cord.

It is my belief that a thoroughly posted, intelligent person is not liable to attempt to simulate such a paralysis. Simulators are usually of a lower order, though frequently possessing a high degree of low cunning. Although the Freeman girl knew enough to simulate loss of sphincter control, her knowledge did not admit of her feigning the areas of anesthesia which should have accompanied a lesion that would destroy the sphincters (it will be remembered that she claimed that her anesthesia extended below the knees). The fact of the sphincter involvement would also militate against the functional form of palsy which might manifest itself by the paralysis of her legs and the stocking form of anesthesia which she simulated. Sphincter paralysis does not occur in hysterical paraplegia. Her case is but an illustration of all of them. They are unable to present a complete picture of the disease they are simulating, whether it is a paralysis in fact or a pathological counterfeit (hysteria).

I can conceive of a bright person quite successfully imitating a hysterical paraplegia, but in these cases, with the examination of the eye fields for the detection of narrow fields for color which are so common in hysteria, the examination of other portions of the body for undiscovered areas of anesthesia, and finally, a search for a sign which I have called attention to and have named hysterical muscular paradox, I feel quite sure that even this simulator would be detected in his simulation, and properly denounced as a fraud.

---

### THE HYDROTHERAPEUTIC TREATMENT OF TYPHOID FEVER.*

I propose to confine my remarks to hydrotherapy, and that particular phase of it which is most valuable.

I think it will be agreed in general by those who are disposed to discuss matters consistently, and certainly by a large proportion of those who are teaching and practicing in hospitals, that hydrotherapy of the bath type is distinctly a proper thing in typhoid fever. Is that, however, the consensus of opinion in the profession as evidenced by their practice? If it is, I am thoroughly mistaken, and my remarks will be superfluous. If it is not, it furnishes a theme of what I shall say. Statistically, this question hardly deserves discussion. The statistics on this subject have grown large, and are overwhelming in the direction of indicating that the typical bath treatment, or the so-called Brand treatment of typhoid, yields decidedly a decreased mortality. Statistics are always deceptive, yet on this point they are so voluminous that we cannot ignore them. It is to be said in fairness to those who oppose the bath treatment that the statistics of it are perfect, and that the statistics of the other forms of expectant treatment are imperfect. In other words, the statistics in favor of the bath treatment, as compiled from various parts of the world, are quite prominent, as compared with the forms of other treatments. However, passing that by as a necessary fallacy that lies in statistics generally, let us consider what we are talking about.

What do we mean by the bath treatment of typhoid fever? Let us take the typical, original Brand bath. It may be defined as the

*By Henry B. Fayvill, M.D., Chicago, Ill.

vigorous rubbing and manipulation of a typhoid patient, well immersed in water of a temperature varying from 60° to 80°. I purposely put it in that form because I wish to emphasize the fact that the manipulation of the bath is an essential part of the process, and that the temperature is subject to variation, it being more a possible source of departure than the manipulation. That is what we mean typically by the bath treatment. What is the theory upon which this treatment is now carried on? Unquestionably, the theory originally with regard to this treatment was that it was an efficient, essential antipyretic measure. Is it now generally so regarded as a primary antipyretic measure? I think it will be agreed by those who have experience in the matter that although the antipyretic value of the bath is great and important, it is a mistake to centralize our attention upon its antipyretic effect. The theory of this treatment is that its effect upon the patient is twofold: first, sedative; second, tonic. Practical observation by everybody who has had experience in this direction will enable them to draw this conclusion, that an adult patient, who is subjected to an intelligent administration of a cold bath in typhoid, typically and regularly, shows marked sedative effects, as manifested, first, by a feeling of comfort, as against a previous feeling of malaise; second, manifested by a tendency to sleep, as contrasted with previous restlessness and inability to sleep. Those conditions are distinctly marked in the ordinary, regular, typical bath.

The second phase of this important condition is said to be tonic. Upon what are we justified in basing that conclusion? First, an improvement in the cerebral condition, a condition of clearness of mind as against hebetude; of alertness as against indifference; of comfort, physical and psychical, as against distress. There is no question that this is a fair index of improved tone. Again, with reference to the digestive tract: first, typically a better digestion, as evidenced in two directions, namely, by the decidedly less typhoid character of the tongue, and by the almost invariable less tendency to meteorism. I do not wish to go outside of the facts in making these statements, but I think they are borne out by experience.

All of these various phenomena incident to the results of baths in typhoid, whether the patients recover or whether they do not, have been ascribed by those who theorize upon this matter to an improved elimination on the part of the patient. In other words, the bath is supposed to operate as a tonic, primarily, in reference to immediate shock; secondarily, as an eliminant of marked power over the individual. Whether this is so or not is inferential. It is based upon obvious improvements in what we have been regarding, I think, and are justified in regarding, as the evidences of toxemia.

Two facts stand together to bear out this idea of better elimination: First, that almost invariably there is a marked improvement, by which I mean an increase in the excretion of urine; that is typical, regular, and reliable. The other is something of which I do not know by experience, but which statistically or experimentally is presented to us, viz., that the toxicity of the urine, after and in connection with the bath treatment, is said to be increased, indicating larger excretion of toxins. Of this there may be some doubt. At any rate, I am not in a position to pass upon it. If, then, this is the theory that underlies these baths—improvements of condition—and if time shall prove this theory to be sound, there can be little doubt that the bath treatment will be placed on solid foundation.

There are, however, modifications of the bath treatment which it is worth our while to discuss. Typically, a patient is taken out of his bed, with a temperature of 103°, 104°, or 105°, as the case may be, and suddenly immersed in a bath of a temperature of 75° or 80°, as the case may be. It is understood by foreign authorities, and some of the best authorities in this country, that this plan yields better results than does any modification thereof. My experience with that form of immersion is so much less than my experience with another that it is with some diffidence I express the opinion that there is a modification of the Brand treatment which in its clinical results is as good, and which in its practical results is much better. In a hospital, for two reasons, a modification is pursued. One is that the actual manual manipulation, the help necessary to immerse patients in the tub, take them out, and dispose of them in that way, is considerable, so much so as to essentially make it almost impracticable in many hospitals. It would not be impracticable, of course, in a large and well regulated hospital. It is not an unfeasible thing to do to administer the Brand treatment, pure and simple, in a hospital.

In my own practice I have departed from the Brand method of treatment, for this reason, which my experience has taught me to believe to be true, namely, that as good results can be obtained by another method, which I will describe in a moment. Private practice demands a modification of the Brand method. The practical affairs of life have to be considered. Therefore, our nurses, who are to be educated for private practice, should be educated in practical ways for it, and it is my opinion that it is as well for us to use a modification in our hospitals. We are all familiar with building in the bed a tub of any kind, either the Burr form or any other form; instead of immersing the patient in water by dropping him in, we pour the water over him, having sufficient assistance to submerge the patient. He is covered with a reasonable amount of water. The water in the tub heats more quickly than in the ordinary bath-tub, and in consequence the measures for cooling the water have to be somewhat different. Where the water is put in at a reasonable temperature, it can be cooled by adding pieces of ice repeatedly until the temperature is reduced to the desired point, and maintained there. My own observation is that a good way is to drop the patient in the tub, remembering in both cases that the friction of the patient is kept up incessantly, which has an important bearing. The patient is kept in the tub for a variable length of time. Most any man who saw for the first time a typhoid patient in a tub of water would say that the patient was being maltreated, for the reason that he becomes cold, shivers, is blue, has distressing symptoms, and frequently implores us to take him out. This, however, is not really the case if we are accustomed to it. It looks like maltreatment, and it takes some experience to go on with it in the face of that; but after all, when a patient is once taken out of such a bath, and rolled in a blanket, he goes immediately into a state of repose, perhaps sleep, and sometimes continues sleeping for hours. That form of bath is practicable in any house, under any circumstances, where there is one person who can devote his attention to the patient, whether it is a trained nurse or not. I say this with emphasis, because it is true, and because the generally prevalent idea is not true. It is of the greatest importance that we come to recognize this can be done in any private house where there are no facilities at all. I do not mean now necessarily the surroundings of opulence.

The great difficulty in having this form of treatment adopted arises from the fact that the majority of patients with typhoid fever recover; hence, all of us are so accustomed to treating them through to recovery, that we expect them to recover without so much fuss. What fuss do we make? As they go on and get sicker and sicker we sponge and pack them; we do all sorts of quasi-therapeutic things to reduce temperature. Does anybody say that sponging or packing is distinctly tonic, sedative or eliminant? It may be, but that is not the idea. The idea of these collateral, alternative procedures is to reduce temperature. But the reduction of temperature is not the point. The condition is the point, and I wish to say without equivocation and with the firmest conviction that the condition incident to the bath treatment is beyond any question better than the condition incident to any other form of hydrotherapy. It is rare in these days in hospital treatment that we see a typical status typhoidus. It is rare that we see a typical, classical typhoid state of the literature. Why? Because, whether the mortality is obviously and noticeably decreased or not, the condition of the patient, the severity of the attack, the general course of the disease, are inexpressibly milder, more manageable, more comfortable under the bath régime than under any other treatment I have ever seen, and I have seen the other in all its horrors. Let us assume, and we will assume a little, that the bath treatment is practicable in private practice with any nurse who is properly trained. It is practicable with anybody who can give his or her attention to it in order to do it.

What are the difficulties in private practice? I will be answered by fifty physicians here to this effect, opposition on the part of the people. Is that a good reason? Obviously, if the treatment of typhoid with the bath is distinctly a better therapeutic measure, the moral obligation is so great that we cannot consider it as a cogent reason. If it is not a good reason, if it is not a sufficient reason, then it is up to us, so to speak, to do the very best we can for our patients. Opposition on the part of the people does not exist. It exists as a remonstrance, as a surprise, as a reaction to an innovation, but it does not exist as an irresistible, immovable obstacle, and it is entirely within the scope of the physician to go in and master the situation.

There are one or two points that I want to speak about. In the early baths, when the patient does not know what is going to happen, you had better not talk about cold tubs. The early bath should be spoken of as a bath. If you wish, give the patient a warm bath. Let the whole process be done with a warm bath until the patient gets over the newness of the situation. Let the first bath be, for instance, not cooler than 90°; the next one reduced to 80°; the third one to 75°, or to the point you desire it. In other words, display a little sense, and there will be no difficulty about it. In the second place, the earliness with which the typhoid régime is instituted and used is as important in the bath treatment as is the antitoxin for diphtheria. It is here we make a mistake. We wait until patients are very ill before we give the tubs. I say this from considerable experience: resort to the use of tubs early; do not wait for a diagnosis, if the temperature warrants the use of tubs. If the case is not typhoid, no harm is done; if it is typhoid, you have doubled and quadrupled your therapeutics.—*Medical Age.*

---

### TREATMENT OF TYPHOID FEVER WITH CASTOR OIL.*

It is proposed in this paper to call the attention of the Association to a treatment for typhoid fever which is at once old and new. The treatment is old in that castor oil has been administered for many years to relieve certain forms of the disease, it being often used to relieve tympanites, constipation and accumulation of undigested and hurtful food. Perhaps there are few physicians present who have

*Read by C. C. Bass, M.D., Columbus, Miss., before the Mississippi Valley Medical Association, in session at Kansas City, Mo., October 15-17, 1902. Published also in *The Virginia Medical Semi-Monthly*.

not used castor oil at some time in the treatment of typhoid fever. It is the equivalent of the established limitative treatment, but it is a better choice of drugs. It is new in that castor oil has never been administered as an exclusive, or nearly exclusive treatment. The quite considerable inquiry which I have made has failed to find the treatment suggested in this light, except in an article of my own, published in the *Mississippi Record*, April, 1902.

This paper will present briefly—first, the theory; second, the method; and third, the result of the treatment.

1. *The Theory.*—Typhoid fever is an acute, contagious, self-limited disease characterized by inflammation, necrosis and ulceration of Peyer's patches and the solitary glands of the small and large intestines. The cases are generally divided into three varieties—the abortive, the mild, and the severe forms. In the abortive forms there is no necrosis and no Peyer's patches. In this form the temperature reaches normal in the second week. This form seldom occurs in the section of the country where I practice. In the mild type, the temperature reaches normal in the third week, with slight elevations generally extending into the fourth week. The bowel lesions may be extensive, but healing is prompt. In the severe form the temperature ranges high. It may reach normal in the fourth week, but may not do so for a number of weeks longer. In the second or third week, the patient passes into a low adynamic condition characterized by a dry tongue, sordes, delirum and muscular disturbances. It is also claimed that severe cases are those in which the absorption of toxins from the alimentary canal is extensive, and the elimination therefrom is defective. In the abortive cases there is seldom tympanites, in the mild ones very little; in the severe ones this is a prominent symptom. The more tympanites, the severer the case, and vice versa. Meteorism arises from two principal causes—first, increased fermentation, which takes place in the intestinal contents; and second, paralysis of the muscular coat resulting from toxemia. If the gases in the tympanitic are not themselves toxic, they, at least, increase the tension in the bowel and increase the absorption of the toxins and the germs that do exist in the canal. It is a firm conviction with me, that the serious nervous and muscular symptoms are, to a very great extent, if not altogether, due to the absorption from the intestinal canal of toxins. The result of the castor oil treatment proves this clearly enough, and, it seems to me, does it conclusively. Take any case of uncomplicated typhoid fever where the temperature ranges above 103 degrees F., and give a dose of castor oil every twelve hours, giving no cther medicine at all, and the temperature will invariably be reduced in three days, and will generally be below 102 degrees F., and always below 103 degrees F. Any case with wild delirium will subside in the same length of time. The effects of the oil is confined to the bowel. It accomplishes what it does effect by eliminating from the bowel germs and toxins which would otherwise be absorbed. If sweeping germs and toxins out of the bowels, thereby preventing their absorption, diminishes these symptoms, then surely the absorption of these poisons must be the thing that causes them or aggravates them. The administration of antiseptics, such as have no effect on the nervous system, will generally accomplish the same thing. This is again evidence that the cause of the severe symptoms, or the cause of their severity, is the taking up of something from the alimentary canal. Therefore the theory is deduced that the more of these gases, toxins and germs that are eliminated from the bowel, the milder will be the case. The thing, then, if this much of the theory is correct, is a drug which will eliminate these poisons from the bowel and, at the same time, has no effect upon the general system, and will not spend the patient's strength. Castor oil is the medicine wanted. It goes through the bowel practically as a bolus, or en masse, and cleanses them more thoroughly than any other purgative, and it does not draw upon the system or any organ for its purgative property, but acts of itself, and is not absorbed to overload the already overburdened blood with abnormal substances. I feel myself justified amply in saying, after repeated and careful tests, that castor oil does not weaken or injure the patient, and that no other purgative known to me or used by me acts in the same way. In typhoid fever, digestion is very imperfect, and fermentation is excessive. We have in the alimentary canal, therefore, at all times, more or less fermenting and undigested food, which, of course, makes a bad application to the inflamed necrosed, or ulcerated mucous membrane. Move this off often enough to prevent any accumulation of it, and protect the membrane with oil, and the inflamed spots will not be likely to pass the necrosis, and ulcers will heal more rapidly. The quantity of this fermenting and undigested food will thereby be lessened, and the case thus held to as mild a course as possible. If the ulcers are slow to heal, and the case runs a long course, the patient is not overburdened with toxins to exhaust him before they do heal and the fever subsides. Abortive cases will be kept abortive, mild cases made milder, and severe cases will be converted into mild ones.

2. *The Medical.*—Taking of tympanites as my guide, my object was to keep the bowels as free as may be of germs, toxins and fermentation When I first began the use of the treatment, I gave one dose of castor oil every twenty-four hours. I found that, after giving a dose, tympanites was partially or wholly relieved, but that it usually began to return before time for the next dose. I also found that the typical typhoid odor of the stool, while perhaps it was partially relieved, was never altogether. I then began to give a dose every twelve hours, and found that in from one to three days these conditions were entirely removed, the stool was rid of the typhoid odor, and was as odorless as that of the healthy man. The presence or absence of the typical odor of the stool became at once another guide in the treatment. These two results—first, the bowels free from tympanites; and second, a stool free from the odor, indicating a satisfactory condition of the canal, can be had by administering a dose of castor oil every twelve hours, but it cannot always be had by less than that.

A dose of castor oil is an indefinite quantity in the treatment of typhoid fever. It varies in different cases, and in different stages and conditions in the same case. It may vary from one to eight drams. Enough to act in three to five hours should be given, but not so much as to act more than twice. If the patient is seen in the first week, when constipation usually exists, the dose will be from two to four drams, but if in the second or third week, when diarrhea is the rule, one or two drams will be the proper dose. After one or two doses have been given, it can be easily regulated. During the second and into the third week, the dose is about the same, but increases considerably during the last week in the bed.

In abortive cases the dose is usually larger all through the course of the disease. The taste is very well disguised by giving it in a warm cup with a little boiled sweet milk.

3. *Results.*—The result of the treatment is about as follows: The temperature ranges lower; the tympanitis and delirium do not occur, and if they are present, they soon subside; diarrhea and dysentery are prevented or checked if they already exist; the disease runs a milder course, and the patient does not lose flesh and strength so fast as he generally dose under any other treatment. The last named result is its greatest advantage over any antiseptic treatment. The explanation is, that any antiseptic that is effective enough to prevent or materially retard fermentation and germ life will also prevent or generally retard digestion. Castor oil does not do this.

Up to the present time, I have treated thirty-two cases of typhoid fever with castor oil, and except in a very few of these cases, in which I gave some other medicine to meet some special symptom, I gave nothing else. In order to be brief, I will report them collectively. Thirteen cases were treated with one dose every twenty-four hours, and nineteen cases with one dose every twelve hours.

Three of the thirteen cases which were treated with a dose every twenty-four hours had a temperature above 103 degrees F., delirium and tympanites when the treatment was begun. These symptoms subsided in from three to five days. The temperature came down below 102½ degrees F. in from two to five days; after that they ran the same mild course that the other ten did. With the exception of one case for one day, and of the three cases in the beginning of the treatment, as just mentioned, the entire thirteen cases had a temperature during their entire course ranging be low 102½ degrees F. Except as mentioned, of the three cases, delirium nor tympanites never occurred. Their temperature reached normal in from fifteen to twenty-six days, and there did not occur a single complication. Of these thirteen cases, four had diarrhea, and one had dysentery, when the treatment was begun. In each instance, these symptoms were entirely absent within four days. Afterwards they continued as the other cases, having one or two actions after each dose of oil.

Of the nineteen cases treated with a dose every twelve hours, there were six cases with delirium, tympanites and a temperature above 103 degrees F. when the treatment was begun. In all of them, those symptoms subsided within three days, the temperature coming below 102 degrees F. There were three other cases in which tympanites was prominent, in which it also subsided. After the third day of the treatment, excepting only in four instances, the temperature in the nineteen cases ranged below 102 degrees F. throughout the entire course. It is to be understood that of the fourteen instances referred to, maybe one, maybe two, maybe three, would occur in a single case. The temperature reached normal in from twelve to twenty-one days from the beginning of the attack. In calculating the duration of the disease, I have not taken the date of taking the bed as the first day of the illness, as is sometimes done, but in each case, inquired carefully as to the first day of the malaise, headache, etc., even when there was no knowledge of the fever, and calculated from that date. One of the nineteen cases should have special mention. After a mild course, the temperature reached normal on the nineteenth day of the illness, and on the twentieth began to rise again, and ran another course of twenty-three days, in which the temperature went above 102 degrees F. several different times. Free hemorrhages occurred on the thirty-first day of the illness, and tympanitis followed administration of opium and withdrawal of the oil. Tympanitis promptly subsided when the oil treatment was resumed. In this case the treatment seemed to fail, the duration of the disease and the hemorrhages considered. Six of these cases had diarrhea when the treatment was begun, which invariably subsided in from one to three days. To sum up the nineteen cases, and leaving out the one specially mentioned, after the third day the temperature ranged below 102 degrees F., tympanites and delirium were absent and the temperature reached normal in from twelve to twenty-one days from the beginning of the attack, making them all abortive or mild cases.

It is true this is too small a number of cases to base positive conclusions on, but it is large enough to commend the treatment to us as worthy of further investigation.—*Medical Times and Register*.

### THE EMPLOYMENT OF DIGITALIS AND ACONITE IN THE TREATMENT OF CARDIAC DISEASE.*

Among all the difficulties which have beset the subject of the proper use of drugs in disease, and there have been many, as we all know, it cannot be doubted that the factor of greatest importance has been the employment of remedies by physicians without their having a correct conception, and sometimes no conception at all, of the pathological process underlying the condition which is to be relieved. This depends upon the fact that many practitioners lack preliminary training, not only in morbid anatomy and morbid physiology or pathology, but also fail to study the possible effect of well-known drugs in abnormal states. The employment of certain remedies in disease has cast discredit upon therapeutics by their abuse, while many physicians who have carefully studied diseased organs become so saturated, so to speak, with the seriousness of the lesions which they find, that they scoff at the thought that drugs can be of service, forgetting that the vital powers are eliminated at the autopsy, and that the conditions present represent a state so grave that death has taken place—that is, the worst possible state of affairs is seen. I have made these opening remarks because I do not wish to be considered a therapeutic optimist or nihilist, and because I so often emphasize the fault of using drugs when they cannot do good that I fear I may be called a therapeutic unbeliever. In no class of cases does what I have said hold true with greater force than in those of cardiac disease. Some physicians are content to diagnose valvular disease, prescribe digitalis, and ignore the state of the heart muscle, the state of the blood-vessels and that of the kidneys, liver, and even the dose of the drug, so long as it is within bounds not poisonous.

It has always seemed to me that it is the duty of the physician to study the condition of the heart muscle, and almost entirely exclude any suppositions as to the condition of the valves of the heart. While this may be an exaggerated way of making the statement which I wish to emphasize, it is resorted to because in the majority of instances we are apt to endeavor to decide which

---

* By H. A. Hare, M.D., Professor of Therapeutics in the Jefferson Medical College, of Philadelphia.

segment is diseased without a correspondingly careful study of the conditions of the ventricular wall.

Again, it is by no means an uncommon practice of physicians, after determining more or less carefully the condition of the heart, to fail to make a careful study of arterial tension, pulse force, and, equally important, to attempt to discover whether there is arteriocapillary fibrosis. Upon the condition of the heart muscle, and upon the development of arteriocapillary fibrosis, much more depends in the diagnosis, prognosis and treatment of a case of so-called cardiac disease than is usually thought. It is also not permissible to reach correct conclusions in regard to these important factors in the case unless at the same time the renal condition is adequately investigated. And, again, it is not sufficient in many of these cases to be content with one or two examinations of the urine, which may fail to reveal albumin, unless at the same time estimations of urea are also made, and a careful record of the quantity of urine and of its specific gravity is kept. Not only do these renal conditions aid us in getting information concerning the probable conditions of the heart muscle and of the blood-vessels, but they also give us an insight into the ability of the kidneys to eliminate poisonous materials and the drugs themselves, both of which, if retained to an abnormal degree, produce results which are disadvantageous.

I have within the last few years devoted a great deal of attention, not only to these factors in these cases, but as to the question of the proper administration of the various cardiac stimulants, and, equally important, as to the dose which each individual patient needs from day to day.

Digitalis, like iron, has proved itself so valuable, doing good in so many instances which seemed grave, that we are wont to forget that, like most things which do good, it can also do harm, and judging from my previous habit, and from the habit of other practitioners, I am convinced that in the great majority of instances digitalis is administered in doses which are much too large, and often continued over a period which is far too long. It is by no means an uncommon thing to find physicians administering as much as ten or even twenty minimums of tincture of digitalis three or four times a day in cases of marked rupture of compensation. There can be no doubt that in some cases such doses are necessary at the beginning of the treatment to meet the crisis which exists, and in much the same way that we are wont to give large doses of mercury in early syphilis, afterward cutting the doses down one half, so it may be necessary at times to give massive doses of digitalis which, after a period, should be rapidly and considerably diminished. I have been surprised to find what excellent results I could produce by the use of such small amounts as one or two minims of an active, physiologically tested tincture of digitalis given three or four times a day, the patient being, of course, required to rest and so give his heart that most needed therapeutic aid when its compensation is ruptured.

Apropos of this, I may add that in my belief we often fail to get results from doses and from drugs upon which we rely, more because we are careless as to the physiological activity of the product than because we have made an error in judgment as to the remedy which is needed, or the dose which is required. With the important subject of the employment of drugs closely related to digitalis in the treatment of various cardiac conditions, there is not space to deal in this paper. In deciding what cardiac stimulant is required in a given case, we must not only consider the condition of the valves and the myocardium as already indicated, but we must, if possible, reach some conclusion in regard to the state of the coronary arteries. Digitalis, which improves the nutrition of the heart, largely by improving the circulation in these arteries, can manifestly do more harm than good if these nutritive vessels are so nearly closed that it is impossible for the heart to pump blood through them in increased quantity. And again, the myocardium may have undergone such advanced degeneration that it is impossible for the digitalis to improve the nutrition of the heart, although at the same time it may be driving the remaining healthy fibers to an endeavor far in excess of their ability.

I am also quite sure that in a certain number of cases of valvular disease the patient does not require digitalis or any other cardiac stimulant for the relief of his cardiac symptoms; but, on the other hand, in addition to rest, will often be greatly benefited by the administration of aconite, which has the same steadying effect upon the heart through its influence on the vagi as has digitalis, while by its sedative influence on the heart muscle in cases of excessive compensation, and by its relaxing effect upon the blood-vessels, it diminishes the overaction of hypertrophy which is sometimes confused with the tumultuous overaction of ruptured compensation. It is much easier for us to conclude, in the case of valvular disease, with dyspnea and disturbed heart action, that these symptoms are due to a failing heart than that they are due to a hypertrophy and an excessive activity. Such cases I have frequently seen in men who are well developed, in the muscular sense, and whose occupation has caused them to do heavy manual work, or to take part actively in some of the severe athletic games. And not infrequently have I seen other cases in which the use of well-balanced doses of aconite and digitalis have produced results which neither drug could produce by itself, although at first glance they are physiological antagonists.

Finally, the utter uselessness of expecting good results from either of these drugs in the treatment of certain cases of myocardium disease which persistently take severe exercise "for their health" needs to be emphasized. I have repeatedly seen cases of men of advanced years with somewhat fibroid blood-vessels who have mistaken the heaviness of advancing years for the heaviness of lack of exercise, and who on the golf field, on the bicycle, or by rowing and walking, have tried to drive away the symptoms from which they suffer, with a result that sooner or later the condition from which they are suffering becomes greatly aggravated, and they become more or less invalids if they are so fortunate as to escape sudden or nearly immediate death from their ill-judged efforts. It seems to me, too, that when we are attempting to treat such cases, and are endeavoring to administer doses and remedies as accurately as possible, we should insist upon quiet and a careful mode of life until we are able to determine that the remedies suit the case, for otherwise the change of exercise or change in diet may not only prevent the remedies from doing good, but also warp our judgment as to our own plan of treatment, and prevent us from instituting it in another case, when in reality, had proper precautions of this kind been taken, we would have increased confidence and been able to do much good to a large class of patients, for it is not to be forgotten that every one in this room, sooner or later, according to his years, his inheritance, and his mode of life, develops more or less arteriocapillary fibrosis, degeneration of his myocardium, and sclerotic changes in his kidneys.

I may close by saying that curiously enough a very large proportion of the patients to which I have recently referred are physicians who, after a long life of intense nervous strain, not infrequently find themselves at a comparatively early age suffering from disorders of the heart, which they fail to recognize, either because on examining this organ they fail to discover murmurs, or because they do not recognize the fact that a physician's life seems to be peculiarly apt, as is that of the banker and large business manager, to develop degenerative cardiac change. The employment of strychnine, belladonna and other drugs, in connection with digitalis and aconite, might be discussed if time permitted, but they are not included in the title of this paper, and therefore cannot be considered.—*The Therapeutic Gazette.*

---

### CANCER.*

Cancer is a disease caused by a peculiar cell-growth called cancer cells. These combined with a liquid substance called the cancerous juice, and is contained in a new fibrous tissue or in the interstices of some other already existing tissue constituents or cancerous growth. Those which form the specific element of cancer are, in their best example, regular spheres. These cancerous cells may deviate from the common type, first through want of development, hence the smallest and least complete cells are found in the quickest growths. In cancers of the soft variety it may be very difficult to distinguish these from fibro-plastic cells. There are a number of varieties of cancer, the type and model of which cancers is commonly called soft encephaloma, or fungous medullaris, and which is known by the large number of cells contained in the meshes of the most delicate fibrous tissue. It is, as its name implies,

*By William Henry, M.D., Harmon, Ill.

of about the consistence of brain. When cut it seems to be of a whitish yellow or pinkish color. On pressure there exudes therefrom a large amount of juice containing an abundance of cell nuclei. A soft cancer forms a tumor of rapid growth, rounded, smooth, more or less lobulated, softer in some parts than others, not tender.

In its earliest stages it may be circumscribed and separated, though it may extend long distances into the interstices of the muscles or other organs; in its later stages it is usually adherent and blended with surrounded parts. If it is near the surface it may, by the rapidity of its increase, cause the skin to ulcerate.

Hard cancer, as its name implies, is of much firmer consistency than the preceding varieties, which is due to the pressure of a firm, dense tissue. In this respect there may be every gradation from a scirrhus as hard as fibro-cartilage to the soft and almost diffluent epithelioma. In well marked cases hard cancer cuts crispy, the cut surface shows irregular interstices, firm, whitish bands, fibrous, with a soft, grayish or yellow matter in the interstices from which the cancerous juice can be made to exude by pressure.

In its outward character it presents itself as a firm, hard, heavy substance, usually nodulated tumor, thus in time the outward parts become adherent and become subject to severe, stabbing pains, lancinating in character ; it distends the skin causing it to break open and form a more or less exuding ulcer, with a peculiar sickening order, with hard, fungous everted edges, with constant sanious discharges and a severe burning pain.

The melenic or black cancer is usually of the soft variety infiltrated with pigment. As a primary disease it usually occurs in the skin about the eye. For example, in a case reported by Mr. Windsor, of Manchester, it was found in the skin, areolar tissue, muscle, pleura, lungs, heart, liver, mesentery, spleen, kidneys, and the uterus.

The colloid, or gelatinous variety of cancer is a peculiar growth composed of a thin membranous or fibrous material, so arranged as to form circular loculi resembling in this respect pulmonary tissue, and filled with globular masses composed of concentric layers of that structureless material which has been described as colloid matter. I saw cases of this variety of cancer that cut through the substance look like clear jelly.

In this variety some have questioned whether it is cancer. Most pathologists class it as such. Lebert describes some, though not all, of the nuclei as having the character of cancer. Its most frequent location, by far, is the peritoneum and abdominal viscera, yet it is found in the female breast and elsewhere. There are many more varieties of the disease than the above mentioned. Hematoid, cystic, osteoid, villous, epithelial and lupus are all of different varieties. What is rose cancer comes under these heads.

Causes of cancer: under this head we are obliged to confess ignorance. It is claimed by some that an injury of the parts is sometimes the cause; neither temperament, mode of life, civilization, previous disease, nor moral influence have proved to be any predisposing cause. Domestic animals are quite as subject to the disease as man. The dark and bilious are not more subject to it than the light and florid. It is said by observers that the rich seem more liable than the poor, but it is claimed that it is because they are not so often cut off by other diseases. The healthy, well-fed, the happy and prosperous, are as liable as their less fortunate brethren.

The disease is said to be more rare in the tropical than in the temperate climates, and although there is no such incompatibility as is supposed between cancer and phthisis is because cancerous patients often display pre-existing tubercular disease, and may become afflicted with phthisis, yet the person may become afflicted with tubercular disease. External violence is not a cause of cancer. Descent from a cancerous parent seems to have some slight influence, and was found by Lebert to exist in about one-seventh of the cases. Sex has some influence. Cancer is from one-third to one-half more prevalent in the female than in the male. Age has some influence, because nearly one-half of the cases occur between 40 and 60 years of age. Cancer is not contagious, as the term cantagion is understood. There were experiments made on dogs by injecting some of the fluid into them. Langenbeck and Lebert by their experiments found cancerous tumors in the bodies of the animals some time after, when they were killed. I have been fortunate—or unfortunate—in the last few years to have seen several cases of cancer in different localities on the body, some of whom went to the so-called cancer doctors, who came home in a short time and were pronounced cured, but after a few months the disease came on with greater violence, suffering terrible torture for months until death only relieved them of their suffering.

The knife I have seen used only to make matters worse. The disease after a short time comes again with greater severity, spreading out in every direction and going to internal organs. I should stop and consider for some time before I would plunge a knife into a cancerous or malignant tumor. I would not consider it good surgery to slash and cut into everything that came along. From the appearance of a cancerous person I cannot think but that it is both a local and constitutional disease; there is a waxy cachexia of the skin. The latest treatment that I have seen is the X-ray upon the cancerous tumor. It is said to destroy it, causing the tumor to disappear.

There is something about the nature of cancer that has not been understood, both as to its pathology and treatment.

A surgeon with his knife may remove a non-malignant tumor which he tcok for cancer and the wound kindly heal, and in his egotism he sits back on his dignity and says that he, with his knife, has taken a cancer and it got well. It was not a malignant tumor, it was only a benign tumor, and he has in his opinion removed a cancer. The cancer specialist with his plasters and poultices puts them on and takes out what he supposed was a cancer, but which was only a benign tumor, which healed, then he heralds it broadcast that he cured a cancer. When he tries it on a cancer, he does not have such good results. Soon it breaks out with greater violence than before.

Now, gentlemen, to sum the whole matter up, as far as known, those who say they cure cancer with plasters or the knife are frauds and deceive the unwary who grab at a straw like a drowning man. I do not here claim but that it yet may be found out.

In a recent article in the *Medical Times*, of New York, taken from the January number, 1902, of the *Journal of the American*

*Medical Association*, Dr. William Allen Pusy gives three cases of undoubted sarcoma treated by him with the X-ray. In one of these cases he says that under the X-ray's exposure, with absolute absence of other treatment, the sarcoma disappeared, and after three months without treatment has shown no evidence of recurrence. In the other two, both operable, the effect on the tumor was negative, although considerable relief from pain seems to have been obtained. The writer thinks that in the first case the definite curative effect of the X-ray on a sarcoma is conclusively demonstrated, and taking it in conjunction with several authenticated cases in the literature is sufficient evidence of its favorable results. He believes that it may be maintained that in cases of sarcoma which cannot be treated in any way successfully by surgical means, the effects of the X-ray should be tried; and further, in cases of sarcoma which have been treated surgically, subsequent use of X-ray exposure as a prophylactic, is a procedure which should be considered.

The above results having been observed by Dr. Charles Warrene Allen, of the city of New York, he was led to adopt the X-ray treatment in his own cases, but not exclusively, as he considered it acts best as an after-cure and prophylactic measure. He has thus far treated nine cases of epithelioma, one upon the chin, two upon the nose, one upon the finger, one upon the forehead and one upon the cheek. Four were recurrent, two cases were primary, one disappeared from observation, the other two did well and might be considered cured, but all are still taking X-ray treatment. Besides these cases he has observed a number of others. He has several times had in consultation a gentleman with an extended growth recurrent, in whose case the X-ray has been practically the only form of treatment applied. He said that the extensive growth had diminished to nearly one-half its size, and the general condition had improved under the X-ray treatment.

In an old gentleman with epithelioma of the nose of many years' standing, which now appears to be cured, the ulceration has healed and the whole surface is desquamating in a manner never before seen after another treatment. Dr. Allen favors the parasitic origin of cancer, in which disease new territory is invaded and involved, much in the same manner as in the diseases of known parasitic origin, good results being likewise obtained in the latter by the X-ray. The belief held by some that it is necessary to produce a so-called burn, he pronounces erroneous. As to the dangers from the X-ray treatment, he believes them to have been overestimated, and that the cases of injury by this method are growing fewer in spite of its much more extensive use.

There seems to be reason in this treatment. It looks very plausible to me that it would destroy parasitic influence upon the cancer, as some aver that it is of parasitic origin, by the use of the X-ray. Time will tell the results upon the disease.—*St. Louis Medical and Surgical Journal.*

---

### SOME FACTS ON ELECTRICITY AS A THERAPEUTIC AGENT.

The therapeutic application of electricity is similar, if not identical, to the therapeutic application of other agents. For the sake of some comparisons we will take mercury. From it we have blue mass, calomel, corrosive sublimate, blue ointment, and other combinations of the same drug, varying somewhat in their degree of action according to the combination used and the susceptibility of the patient. On the other hand, we have the direct, the alternating, the slowly and rapidly interrupted, induced, the sinusoidal, the static, together with the high-frequency, high-tension currents, all electricity, but varying in their physical, physiologic, and therapeutic action, according to the combination used and to the susceptibility of the patient. The combination of the different preparations of mercury is familiar to all of you, but perhaps the expression as applied to electricity will bear some explanation. The working power of electricity consists of a combination of volts, amperes, ohms, time, and when we hear the expression volts, amperes, etc., it means work accomplished. The unit quantity of electricity is called the coulomb, while the ampere is the unit rate of flow. The volt is the unit of pressure. An electric current carrying one coulomb per second is called a current of one ampere.

*Read by F. B. Bishop, M.D., Washington, D. C., before Medical and Surgical Society of the District of Columbia, November 6, 1902.

And a volume of one ampere of current under an E. M. F. of one volt will pass through a resistance of one ohm. Roughly stated, the ohm is the resistance offered by two miles of ordinary copper trolly wire. Again, when a pressure of one volt causes a current of one ampere to pass through a circuit, work is done at the rate of one watt—or 1-746 of a horse-power. This rule holds with all electric currents, whether of high or low pressure, large or small amperage, alternating, direct, or interrupted currents.

With the direct current we have large volume or great amperage and low voltage. The chemic action of this current is very great; while the current from the modern static machine is one of exceedingly small amperage and great voltage—frequently running up into the millions; indeed, the pressure is so great that it is impossible to completely isolate the static current. Between these two extremes comes the induced or faradic current, of moderately high voltage and low amperage.

Thus we can see in prescribing medicine for a patient we must first make our diagnosis and consider the individual peculiarity of the patient, that we may suit the treatment to the case in hand. So in administering electricity we must endeavor to select that current and the modification of that current which, according to our judgment and experience, is best suited to each case. Especially is this so when we consider that with electricity we seek to modify or cure disease by acting directly upon the part affected, or upon the nerves or nerve centers controlling it, and through these upon the blood-vessels or sometimes upon the vessels themselves.

We should remember that it is not the amount of current generated by our apparatus that we must look to, but to the amount actually passing in circuit through the patient at the proper place and in the right direction, and for a length of time sufficient to produce the desired effects. Blood pressure may be relieved, in many cases, and a bounding heart quieted by a moderately strong continuous current, graduated according to the susceptibility of patient. Conversely, the heart may be often stimulated when weak and flabby, and be toned and strengthened by a very mild continuous current, applied over the superior, middle, and inferior cervical ganglia. But as in medicine it must be remembered that what is a weak current for one individual may be strong for another. Some people are especially susceptible to the influence of the galvanic current, so that three or four milliamperes are about all that can be used in the region of the cervical sympathetic ganglia. If we are unable to produce the desired effect with this small amount of current in these patients we are not justified in running our current higher, but must then use it for a longer time, until the amount of work is done.

In giving strong and powerful medicines it is customary to give them highly diluted, that the stomach may be protected and also that absorption may be more certain. So, also, in consequence of the well-known law of conduction and resistance, in order to protect the sensitive skin, which is a very poor conductor, if we wish to give a strong galvanic current we dilute our current, as it were, by increasing the area of our contact electrodes, because "the resistance of a conductor is directly proportional to its length, and inversly proportional to the area of its cross-section." We thereby reduce the irritation to the skin. The electrodes must be well moistened and in perfect contact for all currents of low and medium voltage. The same law of conduction and resistance holds good with the static current; the large, heavy spark is less painful than the light, thin spark, and in giving the Morton wave current the more surface covered by the contact electrode the less the effect is felt by the patient. These large surface electrodes are very necessary when employing the constant (galvanic) current if we wish to influence deep structures, such as the spinal cord, spinal nerves, the sciatic nerve, as well as surface nerves when the nerves or sheaths are congested or inflamed. After an experience of many years with electricity, my impression is that it should be very rarely used merely as a stimulant. This rule applies especially to the treatment of paralyzed muscles. Ordinarily the stimulating effect of electricity is followed by very little reaction, yet we should be very careful not to overstimulate and tire out muscles already weak from having been deprived of their normal supply of nervous energy. Our object should be rather to tone by carefully applied mild currents, gradually increasing the strength as the tone returns.

In this way we will do much good, and at the same time avoid doing harm.

We must not forget that electricity, while flowing, is always doing work. It is our business, therefore, to see that it is working for good, and not for evil. It is not the object of this paper to demonstrate how any individual disease should be treated, but to show that as electricity is a very powerful therapeutic agent and susceptible of doing a great deal of good, it must also be used with intelligence and discrimination if the greatest good is to be accomplished.

When we consider the subject of static electricity we are considering therapeutic possibilities beyond our ken; we are simply in the dreamland of electro-therapeutics. With our modern machines and appliances we are enabled to accumulate enough energy to almost knock one senseless; or the same energy may be so broken up and distributed as to make it seem as light as a morning zephyr and as refreshing as a mountain breeze. While the body may be subjected to a current of millions of volts, the periods per second running well up into the millions renders it absolutely safe. This latter current is known as the high-tension, high-frequency current from the static machine. From what is generally considered physiological reasons, when an alternating current reaches a certain frequency, the painful effect of the current gives way to a sensation almost imperceptible and pleasing. It would seem, however, that we might find physical reasons for this condition. These currents are also subject to the laws previously stated, and to Ohm's law, which states that "the current equals the electro-motive force (voltage), divided by the resistance." Whereas in the galvanic and ordinary faradic current we are dealing with comparatively small potentials, in the static and high-frequency currents the potentials are tremendous, in the step-up transformers used for high-frequency currents.

High-frequency and high-pressure distribute the current so thoroughly that it does not send its full force through a circumscribed area; in fact, the air is full of the current, so that anywhere within the electro-static field a vacuum tube will glow if held in the hand. Here insulation is no longer considered—glass vacuum tubes, wooden rods, hard rubber, and glass rods to a slight degree; in fact, anything will conduct the current—and while a shower of sparks may be obtained, large and small, they are not painful. The air is full of ozone, and everything in the room is more or less charged by induction. The resistance in the circuit is constantly cutting down the amperage, while the voltage is either increased or unchanged. The ordinary disruptive discharge of the static machine is the spark. A brass ball acts as an accumulator, the current remaining on the ball until enough energy has been accumulated, when a considerable charge overcomes the atmospheric resistance of several inches, and jumps to the opposite pole or to any object connected with the earth. A wooden ball of the same size offers so much resistance that the current comes away in a violet or blue discharge, because enough current does not accumulate to form a spark. The same is true of other electrodes of various degrees of conductivity. All these various electrodes cause physical change in the discharge, as manifested by the different colors of the discharge, which in time will find their appropriate therapeutic place.

It can, therefore, be readily seen that a vast field for investigation and great possibilities are open to the static modalities, with their many and varied modifications. We must study our current and the apparatus producing it, as well as the patient and his peculiarities. Some patients are sensitive to the static currents, and are made quite nervous from fear thereby; the same patients may be benefited by the continuous current, and often, after taking treatment with it for a while, will then take one of the static modalities with great benefit. Conversely the static current may sometimes be very beneficially used when the direct current is either not well tolerated or has failed to cure. Therefore it is only by a close and constant study of the various currents as applied to the various individuals that one can hope to gain the greatest measure of success in electro-therapeutics.—*Journal Advanced Therapeutics.*

---

**This Journal and Atlanta Semi-weekly Journal one year for $1.25 cash in advance. No checks. Strictly to new subscribers.**

### CONCERNING ANTIPYRESIS IN CHILDREN.*

The controversy concerning the salutary effect of fevers in the infectious diseases is by no means conclusively ended, but the principle that high temperature must be regulated and controlled is based on substantial clinical grounds, and must be strictly observed in actual practice.

I need scarcely remind you of the great susceptibility of childhood to causes which produce febrile movement. Very slight causes will often produce high fever. The changes in the temperature curve are often very abrupt; and again, there is a disposition to continuous fever in diseases which are decidedly remittent or intermittent in the adult. Altogether children bear fever very well, and almost continuous high fever for weeks, without auto-toxemia, produces very little change in the organism, outside of the disturbances of nutrition. Children differ greatly in their morbid reaction to high temperatures. Some will be very sick with a temperature of 103° F., while others play about with a temperature of 105° F.

It is, therefore, apparent that the urgency of antipyresis depends on the concomitant symptoms, and not on the height of the mercury alone.

It is very questionable whether even moderate temperatures in comparatively mild diseases should be entirely disregarded, if there is evidence of suffering. The parents demand relief, even if no especial effect is produced on the course of the disease by the use of antipyretics. Hence it is the universal practice to administer an antipyretic in all febrile affections. These may even be denounced as harmful, but their use continues.

Of the various antipyretic measures, hydrotherapy certainly takes the first place; but the physician who uses this means to the exclusion of others will find himself seriously handicapped. The physician who has a variety of means at hand and can use them efficiently, will accomplish the best results. The tendency of the disease, the condition of the patient, and the effect of the remedial

---

*Read before the Bethesda Pediatric Society, October 10, 1902, by E. W. Saunders, M.D., St. Louis, Mo., Professor of Pediatrics, Medical Department, Washington University.

agents, must be considered in prescribing a certain treatment. In many cases it is decidedly advantageous to combine internal and external antipyretics. This is especially true in hyperpyrexia. When the temperature is above 105° F., absorption from the alimentary canal is uncertain, and even when drugs enter the circulation their effect is inhibited. In these cases it is well to precede the exhibition of the drug by hydrotherapeutic measures.

This combined method is not sufficiently employed. Some practitioners rely entirely on the administration of antipyretic drugs, while others rigidly hold to the practice of hydriatrics. If the temperature is in the neighborhood of 105° F. the bath should be used until the fever falls several degrees, then an internal antipyretic will exert its physiological effect and maintain the reduction. By using the bath first for some time it is rarely necessary to give heroic doses of the antipyretic drug; moderate doses accomplish our object, and danger of cardiac depression is obviated.

The use of the coal-tar antipyretics is regarded as dangerous, and yet they may be used with safety in many cases, and their analgesic effect gives them an unique position in the treatment of fevers.

In the use of the coal-tar products, it must always be remembered that they depress the activity of those vital processes by which infections are conquered. They should be used very sparingly when there are indications that the patient will need all his vitality to overcome a prolonged bacterial invasion. Their use is also contraindicated in all diseases in which the heart is liable to fail—such as diphtheria and pneumonia.

In the early stages of scarlet fever and the other exanthemata, they are not harmful, and the relief that they afford will generally justify their use. In influenza they are generally indicated for the relief of pain, although some cardiac stimulant should be combined with them. I have found the combination of phenacetin and camphor advantageous. Camphor is a heart stimulant and also nerve sedative; it is also a mild antipyretic, according to Lauder Brunton.

Let me also call attention to the value of pilocarpin as an eliminant and antipyretic in diphtheria and scarlet fever. The salivation produced by this drug is invaluable in preventing toxic lesions, and allows the organism full power to eradicate the disease. When the fever is very high, hydrotherapeutic measures should precede each dose of the drug. I have, for many years, depended largely on pilocarpin and the bath in the treatment of scarlet fever. In diphtheria, as an adjuvant to antitoxin, pilocarpin is almost indispensable; it removes toxin, while antitoxin neutralizes it.

In pneumonia, veratrum viride is the safest antipyretic. It relieves the pain and lowers the temperature, particularly when aided by hydrotherapy, quiets the nervous system and prevents excessive pulmonary engorgement. Its general neglect is undeserved, and authorities who condemn its use are those who have had little or no experience with it. Of course, it should be given under careful supervision, its effect must be closely watched, and when the physiological effect is produced the dose must be reduced or its administration discontinued. Veratrum is one of the best remedies for the relief of headache in the first week of typhoid fever.

Another valuable antipyretic is the external application of guaiacol. DaCosta, in 1884, first proposed this remedy for the reduction of temperature in typhoid fever. He recommended that not more than thirty drops be painted on the abdomen. In a very short time the temperature drops, and profuse sweating ensues. By other clinicians its use has been extended to the treatment of other diseases, particularly tuberculosis and pneumonia. In fact, the lamented Moncorvo proved that the external application of guaiacol can be used as a diagnostic aid to differentiate tuberculosis from malaria, since there is prompt reduction of temperature in the former disease, while in the latter the fever is unaffected.

Just how guaiacol acts in reducing fever is still unsettled. Carter declared that it must act reflexly from its peculiar action on the peripheral nerve. He bases his claim on the ground that the effect is too prompt to allow of effect from absorption. In fact, he could not determine its presence in the urine when the drug was not inhaled.

But to the clinician who watches the fall in the fever and sees the effect on the sudoriferous glands this explanation seems almost ridiculous. The drug must be absorbed, and its different action on the skin and gastroenteric route can be explained in the fact that in the former the liver is circumvented. When given by the mouth it is retained or neutralized in the liver, while it passes directly into the circulation when applied to the skin, and can thus act on the central nervous system.

I have found this method exceedingly valuable as an antipyretic in infants and children. When the stomach is very irritable, and hydrotherapy inapplicable for any reason, the external application of this drug acts well. In pneumonia, particularly, many infants do not bear cold baths well, and here this method is indicated. In typhoid fever, and especially in tuberculosis, there should be no hesitation in applying guaiacol, if the temperature and other symptoms demand its use. With care there is little danger of depression. The susceptibility of infants to this remedy varies greatly; in some, one drop, applied externally, will produce the desired result, in others several drops must be used.

Hydrotherapy is now generally employed, but its practical application demands a knowledge of its principles and also a correct appreciation of a varied technique. The water must be applied differently in different cases. Some children can take the full bath very nicely, others become very chilly and do not react promptly. For general use the wet pack is the most likely to agree.—*Courier of Medicine.*

---

### THE HISTOLOGY OF THE SMALLPOX LESION.*

In view of the prevalence of smallpox in the United States during the last few years there are no details of the disease which are without interest to the profession at large. The busy practitioner has no time to go beyond the clinical features, but it is through the laboratory examination of the finer details that the essential cause and the influences that bear upon it will be given to the world.

* Read before the Clinical and Pathological Section of the Academy of Medicine of Cleveland, October 3, 1902, by Roger G. Perkins, M.D., Cleveland.

The study of the histology and etiology of variola has received widespread attention, and the results so far obtained may be brought into a comparatively brief compass. Early investigators regarded the process as infectious, starting from multiple points in the skin and progressing to a definite acme. At this point the process became reparative in character, until healing was complete. Later observers, among the most prominent of whom was Weigert, considered the starting-point to be the simultaneous necrosis of a number of cells, due to the presence of a toxin, and that the subsequent changes were more or less secondary to this necrosis. The present view is rather in favor of the first suggestion, that the process is infectious rather than toxic.

The majority of the changes occur, in the *stratum mucosum*, just underneath the horny layer, except in those parts of the body in which this last layer is of unusual thickness, as on the palms and soles. In these areas the lesion is almost entirely in this layer.

Among the first changes is a sharply-localized edema, which causes the well-known shot-like hardness of the papules in variola as distinguished from other similar diseases, especially varicella. The cells just underneath the horny layer swell, lose their prickles, and their protoplasm becomes fluid. The nuclei at first show no particular changes, but when the pressure in the cells becomes excessive the walls rupture, the nuclei are extruded and fragment, and the cell-membranes shrink together. The cells in this neighborhood are denser than those lying deeper, as they are undergoing preparation for the horny layer, and the shrunken walls, persist, forming septa, lying for the most part perpendicular to the surface of the skin. These septa divide the lesion into a number of pockets, giving rise to the well-known fact that it is very difficult to evacuate the pock in variola, because a puncture opens only one or two of these small areas.

At this stage the exudate is composed of serum and a large number of plasmà-cells. The presence of these last in quantities at so early a stage is supposed to indicate that the infection is a severe one. There are very few pus cells, and no bacteria.

The cells of the deeper layers do not liquefy, but undergo a peculiar form of degeneration. The cell-body swells and the nuclei divide extensively within it, so that the cells look like small balloons filled with balls, for which reason Unna has given the name of "ballooning degeneration."

It is at this stage that the umbilication so characteristic of variola occurs. The cause has been much in dispute, and there is as yet no absolute agreement on the subject. It is obvious that when there is a hair in the center of the pock the swelling may be restrained in the neighborhood of the hair by direct adhesions, but since it is also true that there may be umbilication without any central hair, and that there may be none even when there is a central hair, this cannot be set down as the essential cause. The presence of the septa above mentioned is probably of no moment as they are so easily destroyed in the subsequent stages. The occurrence of the phenomenon seems rather to be due to the more active swelling of the cells at the periphery of the lesion, where the process may be supposed to be more acute, and to the greater liquefaction at the center, tending to a depression.

This stage reaches its full development at about the sixth or seventh day, and from this point the appearance is markedly different. There is a marked dilatation of the blood-vessels, which have hitherto shown no special changes, and the plasma cells are replaced by the ordinary polymorphonuclear leukocyte or pus-cell. The septa are absorbed and broken down, the umbilication disappears, and a simple pustule is left. From this point the regenerative changes begin. The pustule absorbs and breaks down the overlying horny layer, which has resisted the previous stages, and dries up, forming a scab over the surface. After the formation of the scab the intact epithelium on all sides begins to grow in beneath, in the manner of the closing of an iris diaphragm, finally pushing off the scab and leaving a surface completely covered by epithelium.

In the confluent variety the only essential difference histologically is that pocks are so close together that the septa between them become broken down, forming wider areas of the same type. When the attack is a mild one the papillæ of the true skin are not involved, at least to the extent of necrosis, and the epithelium of the *stratum mucosum* is able to fill all defects. But when the infection is more severe, or when the formation of pus has been stimulated by scratching, there is more or less widespread destruction of these papillæ, and while the epithelium can *cover* the defect, the papillæ are not regenerated and a depressed scar is left.

Though it is practically certain that smallpox is an infectious disease there is as yet no organism discovered which is acknowledged as the cause. There is little doubt that all bacteria which have been described as the cause have been secondary infections, or introduced by errors in technic, and apparently successful inoculations with them have been due to the carrying over of some of the original lymph into the animal under experimentation.

Failure of proof of bacterial origin led to search for some other organism, and observers in England, Germany and Italy described organisms of the group of protozoa. These were not cultivated, and much stress was laid on the appearances in stained sections, the so-called "cell-inclusions." These are very similar in type to the appearances of the same type in malignant tumors which have been fairly conclusively shown to have nothing to do with any parasites. The uniform failure to cultivate the organisms described, and the consequent inability to reproduce the disease with pure cultures, the only proof which can be accepted as final, have prevented the scientific world from receiving these results, but recent literature contains some articles of great interest in this connection.

Funck in Germany, and Ishigami in Japan, claim to have discovered a protozoan in the vesicles of variola and vaccinia, which has a definite life cycle and which can be cultivated. Claim is also made that the cultures of the organism on the special media used are virulent up to the third generation, and the results are almost uniformly successful. If these results are substantiated, they are of immense value, but the failure of confirmation of so many similar claims in other diseases, and especially in malignant diseases, make us skeptical, until additional work, by unprejudiced observers working with the same methods reaches the same results. Until then the verdict must be the same as in the case of malignant diseases—not proven.—*Cleveland Medical Journal.*

---

### THE TREATMENT OF CHRONIC ALCOHOLISM.*

GENTLEMEN:—The man whom I bring before you, admitted to the hospital three weeks ago, is thirty-six years of age, unmarried, and a leather cutter by occupation. His father died at the age of seventy-two of heart disease, his mother at the age of forty-eight, of sciatica, as he states, whatever that may mean. He has a brother and four sisters living and well. The patient had enjoyed fairly good health until two years ago, when he suffered from malaria. He contracted gonorrhea when he was fourteen years of age and had a second attack at the age of nineteen. He has long been addicted to the free use of alcoholic liquors. He has also used tobacco.

Two weeks before his entrance into the hospital he suffered from cough and pain in the limbs. He had recourse to his favorite remedy for all ills, whisky, which he consumed in excessive quantities. During his stay in the hospital he has been constantly incoherent or delirious. The history which we could obtain from him is, therefore, imperfect, and perhaps not altogether reliable. The main fact of gross intemperance, however, is undoubtedly established. He has been able to answer questions, but his replies have often been contradictory. He has had no idea of time or place. Sometimes he fancied that he was in London, sometimes in Dublin, again he would seem conscious that he was in Philadelphia and even, at times, appeared to realize that he was in this hospital.

The diazo-reaction was negative. There were no bacilli tuberculosis in his sputum. Examination of the blood showed 4,300,-000 red corpuscles and 25,500 leucocytes. The urine was straw-colored; alkaline; of sp. gr., 1.010; free from albumin and sugar. There was a flocculent sediment which contained phosphates, calcium oxalate, leucocytes, and bacteria. The blood-vessels of the eye were somewhat congested. When he came into the hospital

---

*Clinical Lecture by John V. Shoemaker, M.D., LL.D., professor of Skin and Venereal Diseases in the Medico-Chirurgical College and Hospital of Philadelphia, delivered in the amphitheater of the Medico-Chirurgical Hospital.

subcrepitant râles were heard all over his chest. He has a slight ascites and his liver is somewhat enlarged. The abdomen is also distended by flatus. Three days ago there was a slight hemorrhage from the stomach.

This melancholy object before us, this wreck of manhood and physical vigor, is one of the innumerable instances of the abuse of liquor. The infrequent drinker, who is easily intoxicated, and even the periodical spreer, with long intervals of abstinence, are in less danger of permanent damage than the man in whose blood alcohol constantly circulates and who, perhaps, is seldom absolutely drunk in the common acceptation of the term. Here we look upon a man in the very prime of life whose system is thoroughly poisoned by alcohol, whose digestive apparatus is disordered, and whose brain is impaired by his brutish indulgence. There are many such in every rank of society.

Alcohol is a direct local irritant, and causes inflammation of the gastric mucous membrane. I have witnessed the most intense acute gastritis as a result of a prolonged drinking-bout. At such times the individuals usually eat but little, and the walls of the stomach are constantly bathed with alcoholic liquid. The more common effect, however, is a chronic inflammation, which spreads to the small intestine, disorders the secretions, impairs appetite, seriously disturbs the digestion. Absorbed into the vessels of the portal circulation alcohol attacks the delicate structure of the liver. That important gland can no longer perform its functions perfectly, and the evils of liver disease are added to those due to derangement of stomach and bowel. In its escape from the body alcohol irritates the kidneys and lays the foundation of chronic Bright's disease. The blood laden with alcohol may excite disease of the arteries, terminating ultimately in aneurism. Upon the nervous system it exerts a peculiarly harmful effect. Neuritis, locomotor ataxia, tremors, palsies, and mental degeneracy are often to be traced to strong drink.

In such a case as that now in our presence the consequences of intestinal autointoxication are to be added to those due to alcohol pure and simple. The decomposition of foods engenders uric acid and other toxic products, which have their own special actions.

The skin and the lungs, as eliminative organs, likewise suffer. The blotched countenance of drunkards is proverbial. Laryngeal and bronchial catarrh is common in this class of people. You will have noticed in this man's history the mention of subcrepitant râles. This is a gloomy picture, gentlemen, and yet I have given you but a slight outline. When filled in and completed it is a black and dismal spectacle, indeed. The moral nature of alcoholics inevitably degenerates, and they transmit unstable natures to their descendants. Vice, insanity, and crime are closely linked.

The natural resistant power of drunkards is markedly lessened, and they often fall victims to infectious diseases, particularly typhoid fever and pneumonia. When they contract typhoid fever they are often affected with wild delirium of the maniacal type. As I have intimated, this patient was carefully examined with reference to possible typhoid fever, but we were able to exclude that disease, and to recognize the case as one of chronic alcoholism without infectious complication.

Pneumonia is generally fatal to people of drinking habits. The records of the Philadelphia Hospital abound with such cases. In a higher sphere of life the effect is the same, and the annals of private practice could add numerous examples to those furnished by public charities.

From what I have said and implied you will form some conception of the various guises which chronic alcoholism assumes in different persons. In some it spends its force principally upon the alimentary canal; in others the liver, the kidneys, the blood-vessels, or nervous tissue is affected. I have now under my care a gentleman afflicted with carotid aneurism as the result of constant tippling; his kidneys are cirrhotic and he suffers from general pruritus. Very often one of the first signs noticed by the patient is that his hand trembles when he begins to write. Another consequence of strong drink is arthritic disease—rheumatism or gout—due to the incapacity of the liver.

It is needless for me to dwell on the fact that the portentous train of ill effects dependent upon the habitual consumption of alcohol may be excited by all varieties of liquors,—those which are brewed from grain, fermented from the grape, and by the distilled spirits. There is some difference among them as to the organs and tissues to which they are most inimical, but the general results exhibit a strong family likeness. The first effect is stimulation, the second is depression. The muscular substance of the heart is gradually weakened by intemperance. This result was very apparent in the case of the unhappy man now in your presence. His pulse has been rapid and compressible.

In the treatment of this case the great objects were to support the heart and promote the activity of the kidneys. When he was admitted to this institution but a small quantity of urine was being secreted. The two indications were filled by the administration of strychnine and digitalis. Strychnine lends strength to the action of the heart and lessens the frequency of its pulsations. The circulation becomes more equitable under its influence, and secretion is promoted. The glandular apparatus of the alimentary canal receives particular benefit. Strychnine is of marked assistance in allaying the gastro-intestinal catarrh which is an almost inevitable and universal result of constant imbibition of alcoholic fluids; it awakens the appetite and promotes digestion. It also relieves the nausea and vomiting to which this class of patients is so subject. The morning sickness of old topers is proverbial. It produces distressing vomiting and retching of an empty stomach. In order to rouse the torpid organ and stimulate an artificial appetite these unfortunate victims have recourse to what is currently known as an "eye-opener," a drachm before breakfast or, to use another old proverbial expression, "a hair of the dog that bit them."

Thus they go on making matters worse and walking in a vicious circle, by which the mischief is continually aggravated. The insomnia and the tremors of chronic alcoholisms are relieved by the use of strychnine, which is furthermore antidotal to the narcotic and toxic effects of strong drink. Strychnine, indeed, is a prominent feature of legitimate and ethical treatment of the assemblage of morbid conditions which are grouped under the comprehensive designation of chronic alcoholism. By supporting the system and satisfying its needs it perhaps is useful, as many believe, in killing the craving for liquor.

For such important reasons and indications this man has had strychnine sulphate as an essential ingredient of his treatment. The drug has been given in the dose $\frac{1}{40}$ grain every four hours, its effects, of course, being carefully watched

He has also been upon digitalis on account of his enfeebled heart and inactive kidneys. There was a time since his admittance when he was passing no more than 16 ounces of urine in the twenty-four hours : a totally inadequate quantity.

The renal sluggishness was decidedly relieved by this agency, and the secretion has been brought up toward the normal standard. He now takes 10 minims of tincture of digitalis with his strychnine, every fourth hour.

Creosote also has been given. This excellent remedy, by virtue of its antiseptic power, checks the fermentation which is the result of the gastro-enteritis, and is instrumental likewise in allaying the inflammation which is such a constant feature of chronic alcoholism.

A hot pack was administered every second day in order to allay the nervous excitement and delirium. That this procedure exercised a beneficial influence was evidenced by the fact that he became quite rational after each application. The effect, it is true, was transitory. The impression of the alcohol upon the cerebral cells had been too powerful and too long continued to be speedily and effectually removed by outward applications, however salutary.

Capsicum is likewise of avail. This remedy increases the appetite and digestive vigor, counteracts nausea, and relieves flatulence. It invigorates the movements of the heart and augments the secretion of urine. These are valuable properties and can often be utilized with advantage in the treatment of such individuals as I have been speaking of to-day. The efficacy of red pepper is, in fact, well known to topers themselves, who are much in the habit of adding it to their "pick-me-ups" after a debauch. Its combination of qualities renders it of service in delirium tremens.

In the management of cases of chronic alcoholism one of the first questions which arises is as regards that of drink. Shall the patient be allowed a regulated quantity of alcoholic liquor, or shall all such indulgence be peremptorily and immediately forbidden? Now, in order to answer this question in the interest of the patient's physical welfare we should consider his actual condition and the manner in which it has been induced. His tissues and cells have long been accustomed to a certain stimulus. The individual has gone on gradually and imperceptibly increasing his doses until every part of his body has become dependent upon the stimulus. If he now falls ill or for any reason comes under the physician's care and if, upon theoretical principles, his allowance of liquor is stopped, the result will be a collapse. These people themselves are often aware of the fact, perhaps as the result of an experience gained during an effort at reformation. "Take me off my booze and I'll die" was the utterance that I once heard from the lips of an old reprobate. Though stated in the slang of the day, there was considerable truth in the belief. We witness this every day in bedridden, febrile, or traumatic cases. Although we know the fountain-head of the disorder is habitual indulgence in drink, yet, if we are wise, we must allow the liquor in order to tide over an emergency. Many and many a time have I seen this fact exemplified in typhoid fever, pneumonia, or other debilitating disease or after an injury. Under these circumstances the patient will do far better when he is allowed a regulated quantity of stimulants, and if they be not permitted he will be likely to die. In all such cases the amount must be lessened gradually, in accordance with the patient's strength and the circumstances of the case. I remember, among many other instances, the case of a man who had been in the habit of drinking a large quantity of liquor every day. His nervous system was badly shattered and he had been advised to abstain entirely from the use of all liquor.

Under this severe rule, however, he became more nervous than ever and his strength failed. I allowed him a moderate quantity of whisky, which is less adulterated than most other alcoholic drinks, and placed him upon strychnine. He soon began to improve. His nerves steadied and he regained his usual muscular power. After a time it was possible to make another reduction in the amount of liquor consumed, and he was restored to comparative health.

As a matter of course, serious structural damage cannot be repaired. We cannot cause cirrhotic organs to return to a condition of integrity. We may succeed, however, in gradually weaning an old toper from his cups and prevent a morbid process from proceeding to extremity.

The prognosis of the present case is comparatively favorable. He is not far advanced in years, and, although he exhibits the initial stage of hepatic cirrhosis, it is possible that, bad as his condition has been and is, we may succeed in arresting the progress of the disease. If he acquires habits of sobriety he may be rewarded by better health than he has possessed for years.—*Medical Bulletin.*

---

### THE USE OF SPLINTS AND COLD IN SCIATICA.

Genuine sciatic neuralgia is not a common condition. Such neuralgias with no marked organic change are rarely seen, and when they are found their treatment is quite uncomplicated, as rest for a few days, anti-rheumatic or gouty medications with local stimulus, either by dry cups or slight blisters, is sufficient as a rule. The sciatica which is seen ordinarily is not a functional disorder, but organic, which exhibits inflammations of sheath, of the connective tissue and of the neurilemma. A better term for this condition is sciatic neuritis.

All physicians recognize the history of these cases. A patient comes to you with recurrent attacks of sciatic pain—until, he states, he cannot stand long or walk without feeling discomfort, generally growing worse towards night. On examination the pain is seen to be worse, especially at the exit notch and where the sciatic nerve pierces the fascia. Other places occasionally are found at which is experienced intense pain, varying in their sites, depending on the position of the inflammation. Different forms of motion seem to affect cases differently; in certain cases standing on the affected leg produces much violent pain at the exit point, so that the man may fall as if he had been struck. Again a backward or forward motion excites pain, or the stretching of the nerve by extension of the leg causes an increase of the pain. Nerve-stretching, if this treatment is carried out, will be unnecessary. It does cure sciatica, but in a medical disease it is better to use medical means.

Fortunately, double sciatic neuritis is very rare, and when it exists it is due to spinal or caudal disease, undoubtedly.

It is first necessary to eliminate all suspicion of rheumatism, syphilis, gout, trauma, or pelvic trouble, reducing the case to one of neuritis, with depressing influences on sleep, appetite, and nutrition. Then comes the application of rest in bed. But it is necessary to do more than that, put the patient's leg at rest in a long splint such as is employed frequently in fractures of the femur. It is better that it extend from the axilla to the foot, kept in position by light bandages. Occasionally it is nice to have it made with a joint at the knee so that it can be fixed rigidly at any angle desired. If cold is to be employed, also a roughly moulded anterior splint, with a wooden attachment carried up laterally to the waist or axilla, or with a wire frame of like shape is required. It is necessary in actual practice to lessen the evils of rest. The ankle must be protected so that the heel does not carry the weight of the leg; it is well to have the knee in gentle flexion, changing it a very little at each dressing of the leg. Later in the treatment at each dressing it is well to flex and extend all the joints slowly and slightly to prevent too great stiffness following. This must be done with caution and care. Bandages only sufficient to keep the splint in place are required, not as much as in a fracture. Occasionally a patient cannot stand a long splint; then an anterior suspension-splint is good in its effects, elevating the limb slightly. The results of this treatment in chronic sciatic pain are excellent, especially in reducing pain. Should this treatment simply ease the patient and not help him out, or if pain-points remain, then a problem arises. Should the patient be well nourished and the case is not an old one, say three or four months old, continue the splint and use daily Paquelin's cautery button on or over the pain-points, being careful not to destroy the skin so deeply as to injure the sensitive epiderm. This is highly recommended by Mitchell. If the case is more ancient or stubborn Mitchell suggests here dry cold, used either with or without absolute rest. Remember that success in medicine depends on attention to details; this is the time for the application of dry cold in sciatic neuritis. An ice-bag kept on the painful nerve-tract day and night for two or three weeks is to be used, moderately filled with ice and water, so as to lie flat.

At the Orthopedic hospital in Philadelphia the leg is placed on a tin or copper gutter, on the under part of which is the ice-case, "three or four inches wide, with a screw opening one and one half inches wide on the side, made to fit the thighs right or left, whilst another at need receives the calf. These vessels have beveled edges at each end. There are openings on the side closed by screw covers, and these cases being filled with ice and water, are then surrounded with some good non-conducting stuff so arranged as to leave a naked space of metal about three or four inches wide over the nerve-trunk. When a thermometer is placed between the calf and the tin box filled with melting ice, the resultant temperature, the product of the skin-temperature and the ice, is about 59° F. It may rise above this, but does not fall below. By using ice in saturated brine with excess of salt, as long as the ice is not melted or the excess of the salt dissolved, the resultant temperature is as low as 46° or 47° F." As the pain disappears and is felt at night or in spots only, lessen the length of use of ice, applying it at night or an hour or two twice daily. Slowly get rid of the splint, and if any pain-points stay use the cautery over them cautiously. Cauteries are not so efficient as massage. Mitchell recommends that muscles of the leg be gently kneaded, without grease, with a gradually deeper surface-rubbing in a downward direction (effleurage), along the line of the nerve. Mitchell's directions to the masseur are as follows: "Let him arrange the patient on his face, with pillows so placed as to be comfortable. Then let him slowly and gently, with the flat hand, extended fingers or heel of the hand, follow the nerve-trunk downward with gentle, deliberate friction, increased in force day by day. A half-hour of this twice a day is desirable. Let him deal with sections of the nerve at a time, with most attention to such as are yet painful, and let this treatment be done without any form of grease until after a time the increasing pressure begins to wear out the skin, when he may use a little vaseline or other like agent."

It is a little troublesome to decide how much exercise the convalescent patient should take. At first he should use crutches, not sitting up long at a time, to prevent pressure on the sensitive nerve.

As to medicines, iron, cod-liver oil and strychnine, if well borne, are good with a mild diet. Strong galvanic currents have been found of service in the milder sciatic neuritis, but generally are painful and less efficient. Later, should the wasted limbs not gain in size and tone, induction currents may be tried. Drugs locally to relieve pain are of doubtful utility.—*Medical Times.*

---

### KERNIG'S SIGN: ITS FREQUENCY OF OCCURRENCE, CAUSATION, AND CLINICAL SIGNIFICANCE.*

According to some writers, Kernig considered his sign as pathognomonic of meningitis, and while most authors indorse his views, some even go further, and say that it is diagnostic of the cerebro-spinal form of the disease.

Rudolph insists that if the sign consists (as Kernig has described it) in inability to extend the knee passively and fully while the thigh is at right angles to the body, then Kernig's sign is present in a large proportion of persons who have not meningitis—in fact he found such an inability present in 60 per cent. of all hospital patients examined—chiefly children suffering from diseases of divers kinds. On the other hand, this sign is not present in healthy children leading active lives.

The sign will be found present in any condition in which the hamstring muscles are in a state of hypertonus. The author found that recumbency is one of the most potent factors in bringing out the sign. A simple method for eliciting this sign is first to extend the knee fully, then flex the thigh on the pelvis and measure the angle at the hip; normally an angle of 90 per cent. should be present.

Kernig's sign is not a special symptom apart from rigidity of the muscles of the body elsewhere, but is the same condition of rigidity shown in the hamstring muscles which we find in the neck, back, abdomen, and upper limbs. Some writers have used the term "Kernig's sign" as applying to this rigidity of the upper limbs.

The sign in meningitis is probably due to irritation of the cere-

* Robert D. Rudolph, in American Medicine, November 8, 1902.

bellum, either by the toxins set up by the meningitis or an extension of the inflammation to the cerebella substance. In those cases of meningitis in which the sign is not present the site of the disease is probably away from the region of the cerebellum. On the other hand, in those in which, after a period of rigidity, flaccidity sets in, probably the cerebellum, having been at first irritated, is then paralyzed by the process, and hence we get the same result as when the cerebellum is experimentally destroyed, i. e., a loss of skeletal tone.—*Maryland Medical Journal.*

---

### DEATH OF MAJOR REED OF YELLOW FEVER FAME.

Major Walter Reed, an officer of the surgeon-general's department of the army, died in Washington on November 23. Death was due to appendicitis, for which an operation was performed on Monday the 17th inst. Major Reed leaves a widow and a daughter residing in this city, and a son, Lieut. W. L. Reed, Tenth Infantry, now in the Philippines. The funeral will take place Tuesday afternoon from St. Thomas's Episcopal Church.

Major Reed was born in Gloucester county, Virginia, in 1851, and was a graduate of the medical department of the University of Virginia and of Bellevue Hospital, New York city. He was appointed an assistant surgeon in the army in 1875, and at the time of his death was first on the list of majors and surgeons in the medical department of the army. He had been known for years as one of the foremost bacteriologists and pathologists of the country. In 1893 he was appointed curator of the Army Medical Museum in Washington, and gave his time to the science which he loved. Combining in an unusual degree scientific accuracy with calm judgment, he was invaluable in his ability to search out the cause of epidemic diseases and trace their progress.

During the Spanish-American war he was a member of the board to investigate typhoid fever in the army. After the war he made several voyages to Cuba and was on duty in Havana, studying the diseases of the island, more particularly yellow fever, as a member of the board to investigate yellow fever. After a series of brilliant experiments, which cost the life of one member of the board early in 1891, it was announced as a proved fact that yellow fever is conveyed by a certain variety of mosquito and introduced into the blood of non-immunes by its bite. Sanitary measures tending to the destruction of the insect and the segregating of infected persons were put into effect immediately in Havana by order of General Wood, with the result that for over a year no case of yellow fever has there developed, though the disease had existed almost permanently in Havana for three centuries.—*Indiana Medical Journal.*

---

### SANITATSRATH.

Dr. C. S. Sewening, of Werther (Westphalia), states (*Deutche Aerztezeitung*, Berlin, October 1, 1902) that the recent numerous communications regarding the employment of creosotal in pneumonia incite him to publish the good results which he has obtained from the drug in some other affections. Amongst others he recounts a case of catarrhal cystitis occurring in a surveyor who had worked for several years in a wet coal mine, and whose urine formed a thick, mucilaginous deposit in the chamber. He was ordered:

Creosotal, 4 grams (1 dram.)

Ol. oliv., 200 grams (6⅔ ozs.)

After he had taken this mixture two or three times a day in tablespoon doses for eight days, his urine became permanently clear.

Another case was that of a young farmer, who came to him about a year ago complaining of a dirty discoloration of the face, hands, etc., being, in fact, a good picture of Addison's disease, Sewening prescribed for him :

Creosotal, 4 grams (1 dram.)
Ol. jecor., 200 grams (6⅔ ozs.)

directing him to take a tablespoonful of the mixture three times daily. After he had taken the medicine twice the spots disappeared, and they have not returned to this day. The man's general condition, also, is perfectly normal.—*Medical Times and Register.*

